# Pimitespib in patients with advanced gastrointestinal stromal tumors in Japan: an expanded access program

**DOI:** 10.1007/s10147-025-02726-0

**Published:** 2025-02-28

**Authors:** Yoichi Naito, Shiro Iwagami, Toshihiko Doi, Tsuyoshi Takahashi, Yukinori Kurokawa

**Affiliations:** 1 Department of General Internal Medicine, Medical Oncology, Experimental Therapeutics, National Cancer Center Hospital East, 6-5-1 Kashiwanoha, Kashiwa-shi, Chiba-ken, Kashiwa, 277-8577 Japan; 2 Department of Gastroenterological Surgery, Graduate School of Life Sciences, Kumamoto University, Kumamoto, Japan; 3 Department of Experimental Therapeutics, National Cancer Center Hospital East, Kashiwa, Japan; 4 Department of Gastroenterological Surgery, Osaka University Graduate School of Medicine, Osaka, Japan; 5 Department of Surgery, National Hospital Organization Kumamoto Medical Center, Kumamoto, Japan

**Keywords:** Antagonists and inhibitors, Compassionate use trials, Gastrointestinal stromal tumors, HSP90 heat-shock proteins, Pimitespib, Japan

## Abstract

**Background:**

Pimitespib, an oral heat shock protein 90 inhibitor, significantly prolonged progression-free survival in patients with advanced gastrointestinal stromal tumors (GIST) in CHAPTER-GIST-301 study. This expanded access program was conducted to evaluate the safety and efficacy of pimitespib in Japanese patients with advanced GIST.

**Methods:**

This multicenter, open-label, single-arm study was conducted in patients (≥ 20 years) with histologically confirmed GIST who had been previously treated with imatinib, sunitinib and regorafenib and had an Eastern Cooperative Oncology Group performance status of 0–1. Patients received pimitespib 160 mg/day for five days, followed by a 2-day rest, in 21-day cycles.

**Results:**

Between February and August 2022, 23 patients were enrolled (median age 59.0 years). Over a median pimitespib treatment duration of 81.0 days, adverse events occurred in 22 patients (95.7%). The most common adverse events were diarrhea (73.9%), nausea (39.1%) and increased blood creatinine (30.4%). Serious adverse events occurred in two patients (tumor hemorrhage and tumor pain); neither was related to pimitespib. One patient had grade 3 diarrhea that was considered treatment-related. Four patients (17.4%) had eye disorders, all of which were grade 1 and treatment-related. The median progression-free survival was 4.2 months (95% confidence interval [CI] 1.9–6.2), the overall response rate was 0% (95% CI 0–16.1) and the disease control rate was 66.7% (95% CI 43.0–85.4).

**Conclusions:**

Pimitespib was well tolerated and effective in patients with advanced GIST in real-world practice in Japan. No new safety signals were identified.

Trial registration: jRCT2031210526 registered 1 February 2022.

**Supplementary Information:**

The online version contains supplementary material available at 10.1007/s10147-025-02726-0.

## Introduction

Gastrointestinal stromal tumors (GIST) are a group of sarcomas that develop primarily from the precursors of interstitial cells of Cajal, the cells responsible for peristalsis [1]. The incidence of GIST ranges from 4 to 22 cases per million per year [1, 2]. Mutations in v-kit Hardy-Zuckerman 4 feline sarcoma viral oncogene homolog (KIT) and platelet-derived growth factor receptor alpha (PDGFRA) genes, both of which encode receptor tyrosine kinases, are the most common oncodrivers responsible for the development of GIST and are found in 60–85% and 10–15% of these tumors, respectively [1, 3–5]. Tyrosine kinase inhibitors (TKIs) are the mainstay of treatment for advanced GIST [1]. Imatinib, sunitinib and regorafenib are recommended as first-, second- and third-line treatments, respectively [6–8]. Recent developments in the management of advanced GIST include the approval of ripretinib for fourth-line treatment [9] and avapritinib for GIST harboring a PDGFRA exon 18 mutation, such as the PDGFRA D842V mutation [10], which confers primary resistance to imatinib and is found in approximately 8% of tumors [5]. However, neither of these TKIs are approved in Japan [11]. Furthermore, many patients develop secondary resistance to TKIs as a result of acquired mutations in KIT and PDGFRA [5, 12]. For example, 40–50% of patients treated with imatinib develop secondary resistance within two years [12]. Therefore, new treatment options with different mechanisms of action are needed to improve outcomes in patients with advanced GIST.

Pimitespib, an oral heat shock protein 90 (HSP90) inhibitor that selectively binds to HSP90α and HSP90β, is one such option [13]. HSP90 is a molecular chaperone that plays an essential role in stabilizing KIT and PDGFRA proteins [14]. Pimitespib induced apoptosis in both imatinib-sensitive and -resistant GIST cell lines and inhibited their growth [15]. In June 2022, pimitespib was approved in Japan as a fourth-line treatment for GIST and is now recommended by the Japan Society of Clinical Oncology’s GIST clinical practice guidelines [11, 16].

Approval of pimitespib for GIST in Japan was based on the results of the pivotal CHAPTER-GIST-301 study, in which fourth-line treatment with pimitespib significantly extended progression-free survival (PFS) (2.8 vs 1.4 months; hazard ratio 0.51; one-sided p = 0.006) compared to placebo in patients refractory or intolerant to standard therapy [17]. Pimitespib demonstrated a distinct safety profile compared with other drugs used for GIST. Diarrhea was the most common adverse event (AE) (74.1%) [17]. Most of the reported eye disorders, which are characteristic of HSP90 inhibitors [18], were of grade 1 severity. Overall, the incidence of treatment-related AEs (TRAEs) such as rash and hand-foot syndrome, was lower with pimitespib 17 than with TKIs used in advanced GIST [19–23]. Pimitespib also had efficacy in patients with various types of KIT mutations [17].

Before pimitespib received marketing authorization in Japan, an expanded access program was undertaken to provide treatment opportunities for patients with advanced GIST who had already received standard treatment and to further evaluate its safety and efficacy. The results of this study are reported here.

## Patients and methods

### Study design and participants

This multicenter, open-label, single-arm, expanded access program was conducted to assess the safety and efficacy of pimitespib in Japanese patients with advanced GIST (jRCT2031210526).

This study used the same inclusion criteria as the CHAPTER-GIST-301 study [17]. Patients were eligible if they were ≥ 20 years old; had a histologically confirmed diagnosis of GIST; had been treated with imatinib, sunitinib and regorafenib previously; had clinical progressive disease (PD) or radiological PD (according to Response Evaluation Criteria in Solid Tumors [RECIST] version 1.1) on the most recent treatment or were intolerant to this treatment; had ≥ 1 measurable lesion according to RECIST version 1.1 (except lymph nodes, which were considered to be non-target lesions, regardless of size); and had an Eastern Cooperative Oncology Group (ECOG) performance status of 0–1. A key exclusion criterion included corrected visual acuity < 0.5 for both eyes. Patients who received pimitespib for GIST in a phase 2 study or in the CHAPTER-GIST-301 study and did not have primary PD or grade ≥ 3 AEs related to pimitespib could enter the present study if they fulfilled the remaining inclusion criteria [17, 24].

### Ethics

This study was conducted in accordance with Good Clinical Practice and, after pimitespib received marketing approval, Good Post-Marketing Study Practice, the ethical principles of the Declaration of Helsinki, and applicable regulatory requirements. The protocol and its amendments were approved by institutional review boards before the study began. All patients provided written informed consent.

### Procedures

Patients received pimitespib once daily, orally and on an empty stomach, for five consecutive days, followed by a 2-day rest, in 21-day cycles. The starting dose was 160 mg/day. Patients who transitioned from previous studies (the phase 2 study or the CHAPTER-GIST-301 study) continued on the same dose they had previously received. Pimitespib interruptions of up to 21 days and dose reductions of up to three dose levels (120, 80 and 40 mg/day) were allowed to manage AEs. If an interruption of > 21 days was required, pimitespib was discontinued. Other criteria for discontinuation of pimitespib were PD, withdrawal of consent, occurrence of intolerable AEs and commercial availability of pimitespib to the individual patient.

Safety data were collected from the first dose of pimitespib administered in this study until 30 days after the last dose (safety follow-up). However, AEs considered by the investigator to be related to pimitespib reported > 30 days after the last dose were also recorded. Important identified risks of pimitespib were severe diarrhea, eye disorders and hemorrhage. In this study, severe diarrhea was defined as grade 3 or higher diarrhea. Vision abnormalities of grade ≥ 2 severity were considered to be AEs of special interest. AEs were graded according to the Common Terminology Criteria for Adverse Events (CTCAE), version 4.03.

Tumor assessments were conducted using computed tomography (CT), magnetic resonance imaging (MRI) or X-ray, and were based on RECIST version 1.1. Tumor assessments were performed at baseline and every 8–9 weeks in patients who transitioned from previous studies.

### Outcomes

The primary objective was to evaluate the safety of pimitespib in patients with GIST. Safety endpoints were the incidence of AEs, TRAEs, changes in vital signs, laboratory tests and electrocardiography.

The secondary objective was to evaluate the efficacy of pimitespib. Efficacy endpoints were PFS, overall response rate (ORR) and disease control rate (DCR). PFS was defined as the period from enrolment until radiological PD or death. ORR was defined as the proportion of patients whose best overall response was complete response (CR) or partial response (PR). DCR was defined as the proportion of patients whose best overall response was CR, PR, or stable disease (SD). For transitioned patients from previous studies, baseline tumor assessments at those studies were used.

### Statistical analysis

The planned number of enrolled patients was 30, to be recruited from three institutions in Japan. No formal sample size calculation was performed.

Safety was analyzed in all patients who received ≥ 1 dose of pimitespib (all-treated population, ATP). Efficacy was analyzed in patients who had ≥ 1 tumor assessment after the start of pimitespib treatment but excluding patients who transitioned from the phase 2 study or the CHAPTER-GIST-301 study (full analysis set, FAS).

Descriptive statistics were used for most data, with the number and proportion of patients estimated for categorical variables, and medians and ranges estimated for continuous variables. PFS was estimated using the Kaplan–Meier method and presented as medians and their associated 95% confidence intervals (CI).

PFS was also estimated by pimitespib dose reduction and baseline patient characteristics in subgroups of patients (ECOG performance status, previous lines of anticancer therapy, age, liver metastasis, peritoneal metastasis, and primary tumor type) using the Kaplan–Meier method.

Statistical analyses were conducted using SAS, version 9.4, and SAS/STAT, version 15.2 (SAS Institute, Cary, North Carolina, USA).

## Results

### Trial profile and patient characteristics

Between February and August 2022, 23 patients were enrolled at three institutions, received pimitespib, and were included in the ATP. Two patients transitioned from the CHAPTER-GIST-301 study and none transitioned from the phase 2 study. Therefore, the FAS comprised 21 patients.

The median age of patients in the ATP was 59.0 years. Twelve patients (52.2%) had an ECOG performance status of 0, and 11 patients (47.8%) had a status of 1. All patients had received treatment for advanced or metastatic disease before entering this study, including 21 patients (91.3%) who had received imatinib for advanced/metastatic disease (two patients [8.7%] had received imatinib as adjuvant therapy) and 23 patients (100.0%) who had received sunitinib and regorafenib. Most patients had received multiple lines of anticancer treatment; eight patients (34.8%) had received three lines while 15 patients (56.5%) had received four or more lines (Table 1). The FAS had a similar patient background (Supplementary Table 1). In this study, genomic testing was not carried out, but known genomic information was collected for 10 of 23 patients. *KIT* exon 9 mutation was present in 2 patients, *KIT* exon11 mutation was present in 5 patients, and *PDGFRA* mutation was present in 1 patient.

**Table 1:** Baseline patient characteristics in the all-treated population

	ATP (n = 23)
Age, years, median (range)	59.0 (32–77)
Sex, n (%)	
Male	15 (65.2)
Female	8 (34.8)
ECOG performance status, n (%)	
0	12 (52.2)
1	11 (47.8)
Primary tumor, n (%)	
No	17 (73.9)
Yes	6 (26.1)
Primary tumor site, n (%)	
Small intestine	13 (56.5)
Stomach	9 (39.1)
Other	1 (4.3)
Metastases site, n (%)	
Liver	14 (60.9)
Peritoneum	17 (73.9)
Surgery for primary disease, n (%)	19 (82.6)
Therapy for advanced/metastatic disease, n (%)	
Imatinib	21 (91.3)
Sunitinib	23 (100.0)
Regorafenib	23 (100.0)
Other	4 (17.4)
Number of prior systemic anticancer therapies, n (%)	
3	8 (34.8)
4	7 (30.4)
5	6 (26.1)
6	1 (4.3)
≥ 7	1 (4.3)

*ATP* all-treated population, *ECOG* Eastern Cooperative Oncology Group

Regarding the two patients transferred from the CHAPTER-GIST-301 trial, both had been receiving pimitespib for more than 550 days. One patient experienced the adverse events of diarrhea (grade 1), alopecia (grade 1), malaise (grade 1), abdominal pain (grade 2), nausea (grade 1), and anemia (grade 2), while the other patient experienced the adverse events of paronychia (grade 2), hypertension (grade 2), and abdominal pain (grade 2). Neither patient had the *KIT* exon 9/ 11 mutation.

All patients completed the study treatment by November 2022. The reasons for discontinuation were disease progression (n = 13), pimitespib becoming commercially available (n = 9) and physician’s decision (n = 1). There were no discontinuations due to AEs.

The median duration of pimitespib treatment was 81.0 (range: 11–166; Fig. 1) days, excluding the treatment duration in the CHAPTER-GIST-301 study (n = 2) and after pimitespib became commercially available (n = 9).

**Fig. 1:**
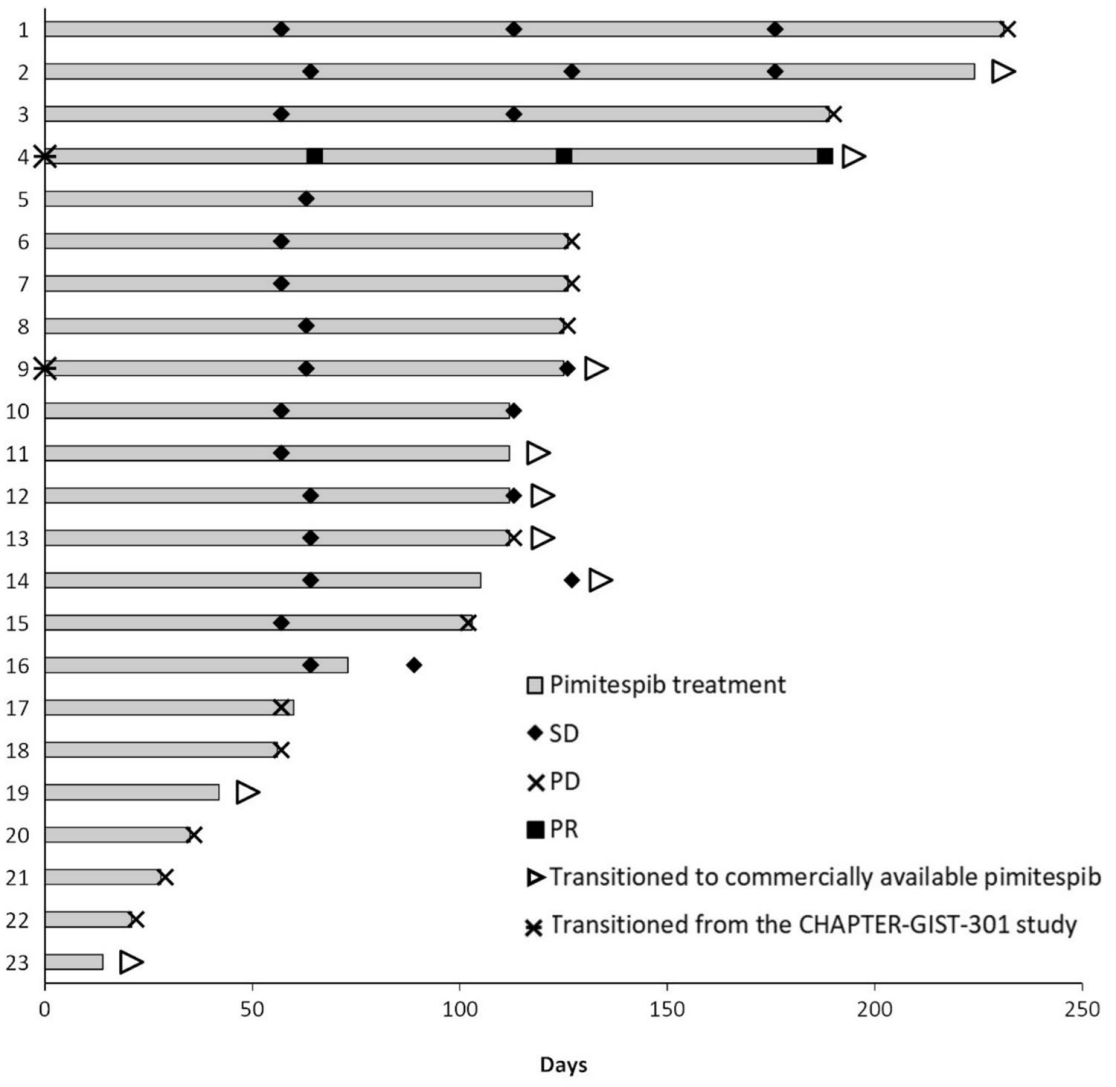
Duration of treatment in the all-treated population (n = 23). *PD* progressive disease, *PR* partial response, *SD* stable disease

### Safety

Twenty-two patients (95.7%) experienced an AE. The most common AEs were diarrhea (73.9%) (Table 2).

**Table 2:** Summary of adverse events in the all-treated population (n = 23)

n (%)	AEs^a,b^		TRAEs^a^	
	Any grade	Grade ≥ 3	Any grade	Grade ≥ 3
Any	22 (95.7)	3 (13.0)	20 (87.0)	2 (8.7)
AEs occurring in ≥ 10% of patients				
Diarrhea	17 (73.9)	1 (4.3)	17 (73.9)	1 (4.3)
Nausea	9 (39.1)	–	9 (39.1)	–
Blood creatinine increased	7 (30.4)	–	7 (30.4)	–
Anemia	6 (26.1)	2 (8.7)	1 (4.3)	–
Malaise	5 (21.7)	–	4 (17.4)	–
Night blindness	3 (13.0)	–	3 (13.0)	–

*AE* adverse event, *MedDRA* Medical Dictionary for Regulatory Activities, *TRAE* treatment-related adverse event

^a^AEs were classified using MedDRA, version 25.1

^b^AEs of any grade that occurred in ≥ 10% of patients

Serious AEs (SAE) occurred in two patients (8.7%): grade 3 tumor hemorrhage (resolved after pimitespib interruption) and grade 2 tumor pain (resolved without any changes to pimitespib). Neither SAE was considered to be related to pimitespib.

Several patients had AEs that were among the important identified risks of pimitespib (i.e. severe diarrhea, eye disorders and hemorrhage). Regarding grade 3 diarrhea, fter initial dose reduction and then interruption of pimitespib, this AE was resolving at last follow-up. AEs classified as eye disorders occurred in four patients (17.4%), including night blindness in three patients (13.0%) and blurred vision in one patient (4.3%). All AEs classified as eye disorders were grade 1 severity, considered to be treatment-related and did not require dose modifications. One patient had grade 2 tumor hemorrhage, not requiring dose modifications, and another had grade 3 tumor hemorrhage, requiring interruption. Neither of these AEs was considered to be treatment-related.

No AEs resulted in death or led to discontinuation of pimitespib. AEs that led to dose reduction occurred in five patients (21.7%). Of these, diarrhea (n = 3, 13.0%), increased blood creatinine (n = 2, 8.7%) and nausea (n = 2, 8.7%) occurred in two or more patients. AEs that led to dose interruption occurred in 11 patients (47.8%). Of these, diarrhea (n = 5, 21.7%), increased blood creatinine (n = 3, 13.0%) and nausea (n = 2, 8.7%) occurred in two or more patients. No AEs of special interest (grade ≥ 2 vision disorders) occurred. There were no new adverse events in the two patients transferred from the CHAPTER-GIST-301 study.

### Efficacy

Efficacy was assessed in 21 patients included in the FAS.

**Fig. 2:**
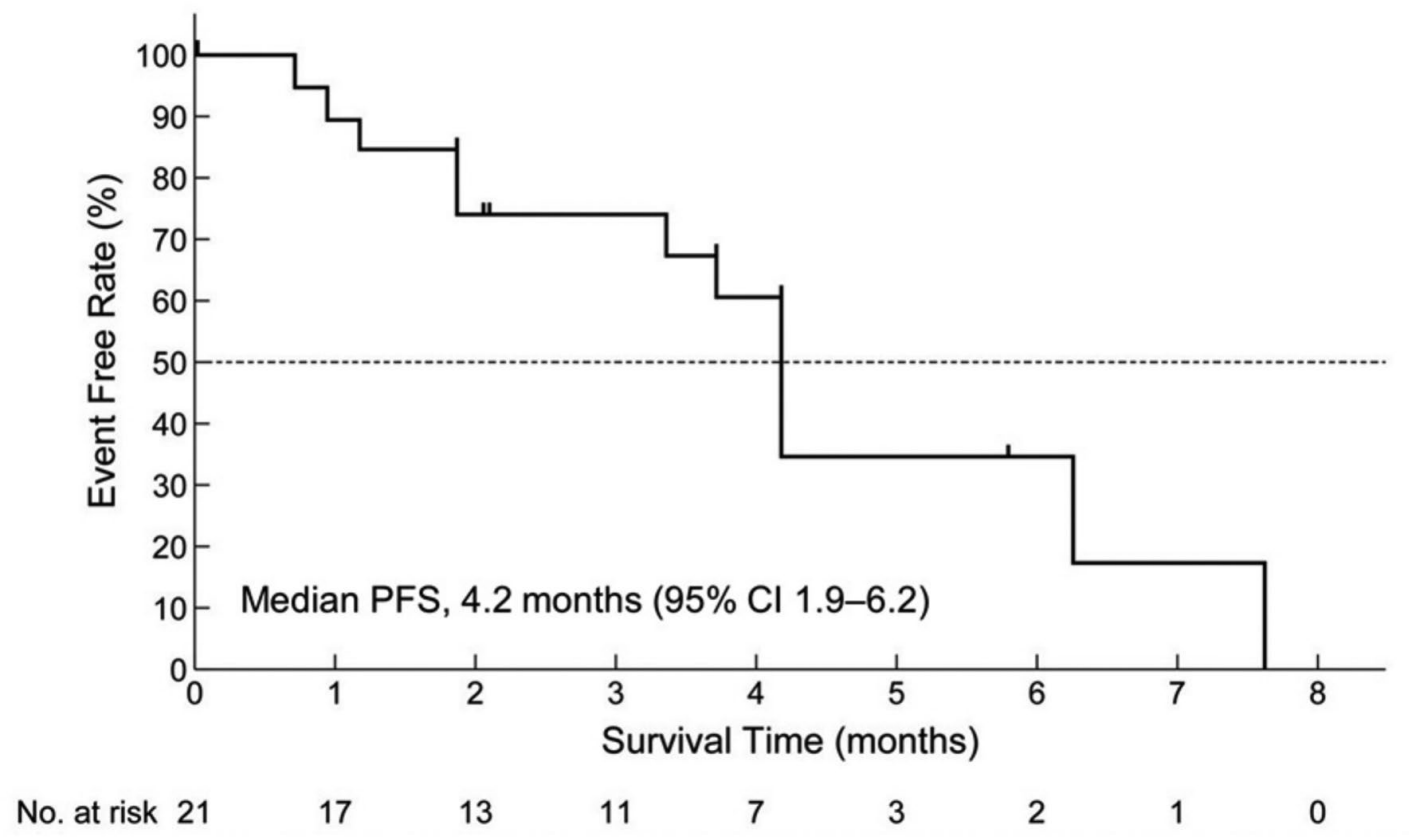
Kaplan–Meier analysis of progression-free survival in the full analysis set (n = 21). *CI* confidence interval, *PFS* progression-free survival

PFS events occurred in 12 patients (57.1%). The median PFS was 4.2 months (95% CI 1.9–6.2; Fig. 2). There were no significant differences in PFS in subgroups of patients categorized according to ECOG performance status (0 vs 1), number of previously received anticancer therapies (3 vs ≥ 4), age (< 65 vs ≥ 65 years), or pimitespib dose reduction (yes vs no; Supplementary Table 2). However, the number of patients in each subgroup was small.

**Fig. 3:**
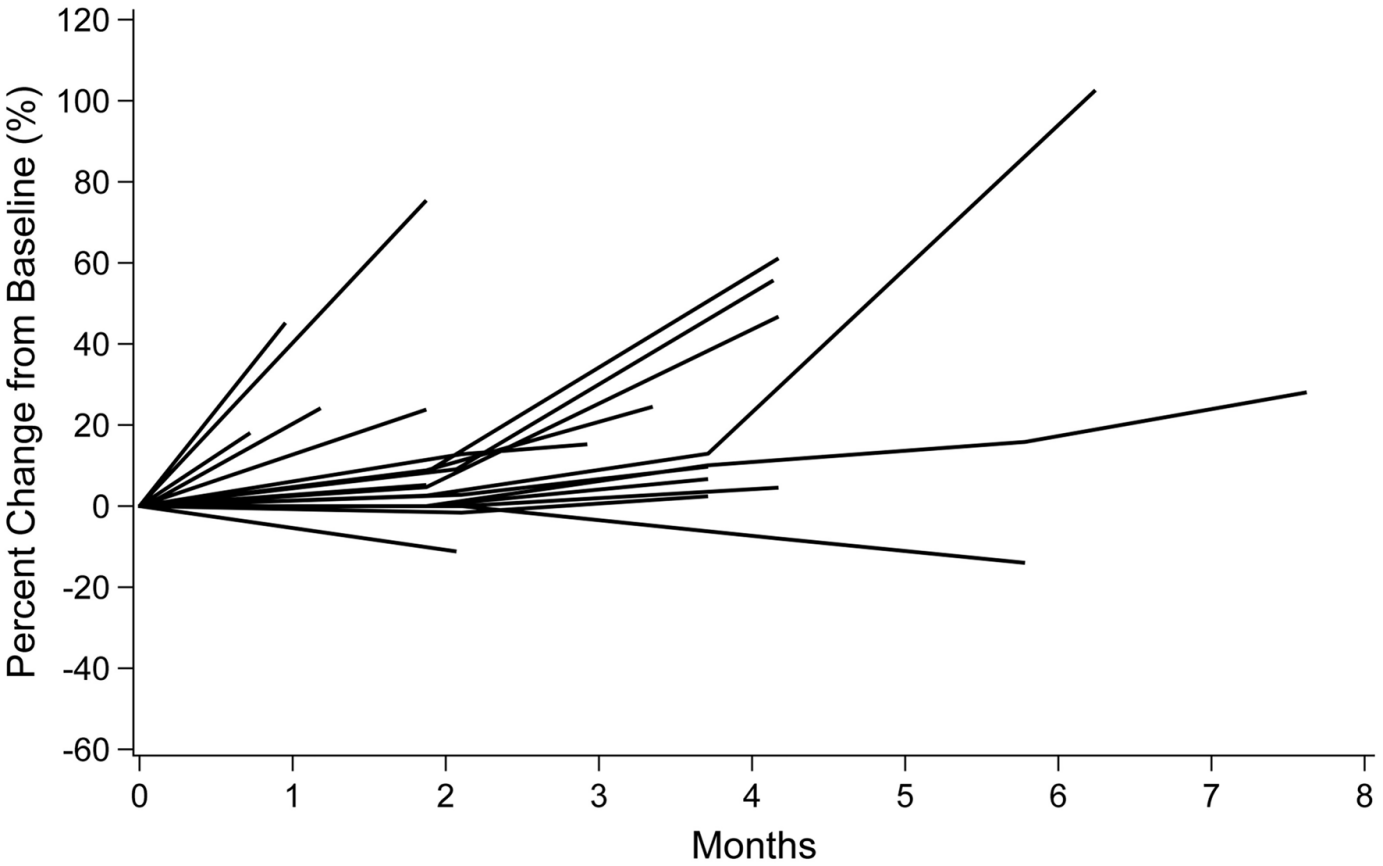
Spider plot of tumor shrinkage in the full analysis set (n = 21)

None of the patients in the FAS had CR or PR. Fourteen patients (66.7%) had SD and five patients (23.8%) had PD; response could not be evaluated in two patients (9.5%). The ORR was 0% (95% CI 0–16.1) and the DCR was 66.7% (95% CI 43.0–85.4). Tumor shrinkage of approximately 10% was observed in two patients (Fig. 3).

After completing treatment with pimitespib, 15 patients (71.4%) received subsequent anticancer treatment. Of note, data on subsequent treatments were not collected after the end of safety follow-up. Among these, 14 received systemic drug therapies and one received radiotherapy. Systemic drug therapies included commercially available pimitespib (n = 6), imatinib (n = 4), sunitinib (n = 1) and regorafenib (n = 3). Of the eight patients in the FAS for whom pimitespib became available commercially, six patients continued treatment, while two patients were lost to follow-up due to transfer to another hospital.

The two patients who transitioned from the CHAPTER-GIST-301 study received pimitespib as sixth-line treatment for advanced GIST, and continued pimitespib treatment after this expanded access program. One of these patients received 55 cycles (49 in the CHAPTER-GIST-301 and six in this study), over 37 months, while the other received 33 cycles (24 in the CHAPTER-GIST-301 and nine in this study), over 22 months.

## Discussion

This multicenter, open-label, single-arm, expanded access program evaluated the safety and efficacy of fourth-line or later pimitespib treatment in patients with GIST who had previously received imatinib, sunitinib and regorafenib. As the numbers of patients administered pimitespib in the previous Phase 2 and 3 studies for patients with GIST were not large, further data on safety and efficacy have been accumulated. In the patients transferred from the CHAPTER-GIST-301 study, we were able to evaluate delayed adverse events with long-term administration. The results of the study show that pimitespib was well tolerated and effective in this heavily pre-treated population.

The safety and efficacy of pimitespib in patients with advanced GIST in Japan were previously evaluated in the single-arm phase 2 and in the randomized, placebo-controlled phase 3 CHAPTER-GIST-301 studies [17, 24]. Although all three studies used similar eligibility criteria, baseline patient characteristics differed. A higher proportion of patients in this study received four or more prior lines of treatment than in the phase 2 and CHAPTER-GIST-301 studies (56.5% vs 52.5% and 31.0%). Additionally, a higher proportion of patients in this study had ECOG performance status of 1 (47.8% vs 30.0% and 15.5%) [17, 24]. In Japan, there was no standard treatment for fourth-line treatment such as ripretinib until pimitespib was approved, and there were no clinical trials targeting GIST patients receiving fourth-line or later treatment. Therefore, it is possible that patients who had their ECOG PS worsen after standard treatment or received other treatments such as imatinib rechallenge were more likely to be included in this study. By comparison, our study population had similar ECOG performance status to that of a real-world study of regorafenib in Japan, with 52.2% vs 48.5% having an ECOG performance status of 0, and 47.8% vs 45.5%, having a status of 1 [25].

The safety profile of pimitespib in this study was generally consistent with previous Japanese studies, despite conditions more closely approximating real-world clinical practice compared with the CHAPTER-GIST-301 study. While the method of collecting safety and tolerability data in this study was the same as in the CHAPTER-GIST-301 study, the incidence of grade ≥ 3 TRAEs was lower in this study than in the phase 2 or CHAPTER-GIST-301 studies (8.7% vs 52.5% and 25.9%) [17, 24]. One reason may be that this study was conducted at the same sites as the CHAPTER-GIST-301 study, leading to better toxicity management due to increased experience with pimitespib. Diarrhea was the most common TRAE in all three studies, occurring in 73.9% of patients in this study and in 80.0% and 74.1% of patients in the phase 2 and CHAPTER-GIST-301 studies, respectively [17, 24]. Treatment-related nausea was reported more often in this study than in CHAPTER-GIST-301 (39.1% vs 24.1%) and at approximately the same incidence as in the phase 2 study (40.0%) [17, 24]. All cases of treatment-related nausea reported in this study and CHAPTER-GIST-301 were of grade 1 or 2 severity, while two grade ≥ 3 cases occurred in the phase 2 study [17, 24]. No serious AEs occurred during this study and no new safety concerns were identified, including in the two patients who transitioned from the CHAPTER-GIST-301 study.

Eye disorders were reported in 17.4% of patients. All AEs classified as eye disorders were of grade 1 severity and none required pimitespib dose modifications. Eye disorders were reported in 20.0% of patients in the phase 2 study 24 and in 27.6% of patients in CHAPTER-GIST-301 (data on file). HSP90 plays multiple roles in the physiology of the retina and eye disorders are often reported with HSP90 inhibitors [18].

The efficacy of pimitespib was evaluated on the basis of the data collected solely during this study, excluding previous treatment during CHAPTER-GIST-301 and subsequent treatment with commercially available pimitespib. The median PFS was favorable in this study compared with CHAPTER-GIST-301 (4.2 vs 2.8 months) and similar to that observed in the phase 2 study (4.4 months) [17, 24]. This may be due to differences in pimitespib treatment duration in each study. The median treatment duration in this study was 81.0 days, which did not include treatment with commercially available pimitespib, compared with 77.5 and 60.0 days in the phase 2 and CHAPTER-GIST-301 studies, respectively [17, 24]. The findings from this study also compare favorably with the median PFS observed with imatinib rechallenge (1.8 months) in a randomized, placebo-controlled trial in GIST patients previously treated with imatinib and sunitinib [26]. Therefore, the results of this study suggests that the efficacy of pimitespib observed in clinical trials may be reflected in real-world practice.

Ripretinib, another novel treatment recently approved for advanced GIST in the USA, has not been studied in Japanese patients and is not yet available in Japan [11]. Ripretinib is a type II switch-control TKI designed to address the various KIT and PDGFRA mutations found in GIST [27]. Although a network meta-analysis has been conducted to analyze the outcomes of phase 3 studies for the treatment of third-line or beyond GIST, the results of which suggested that pimitespib has one of the most tolerable toxicity profiles [28], further research is necessary to clarify the place of both pimitespib and ripretinib in the management of GIST. Pimitespib (alone and in combination with imatinib) is being evaluated as second-line treatment in patients with GIST refractory to imatinib in the ongoing CHAPTER-GIST-101 study (NCT05245968) [29].

This study provides valuable insights into the potential of pimitespib for advanced GIST treatment. Nevertheless, it has several limitations that warrant mention. Firstly, its open-label, single-arm design may have introduced bias, particularly as efficacy evaluations were conducted by the investigator, without the involvement of an independent review board. Furthermore, the sample size was relatively small, which may limit the generalizability of the results. Geographically, the study was limited to Japanese patients, limiting the global applicability of the findings. In addition, the absence of genomic analysis means that the efficacy of pimitespib in relation to KIT mutation status remains unknown. Finally, no data were collected regarding survival status post-study or subsequent exposure to commercial pimitespib. This results in a lack of long-term safety and efficacy data, which is particularly relevant as these findings may differ with extended treatment duration.

In conclusion, the results of this study show that pimitespib was well tolerated and effective in patients with advanced GIST in Japan. These findings support the safety profile demonstrated by pimitespib in the phase 2 and CHAPTER-GIST-301 studies, with no new safety concerns identified.

## Supplementary Information

Below is the link to the electronic supplementary material.

Supplementary file1 (DOCX 32 KB)

## Data Availability

The data will not be shared in accordance with the sponsor’s data sharing policy, as this is a small study. The sponsor’s data sharing policy can be found at https://www.taiho.co.jp/en/science/policy/clinical_trial_information_disclosure_policy/index.html.”
