# Supplementary material for: Pimitespib in patients with advanced gastrointestinal stromal tumors in Japan: an expanded access program
Source: Int J Clin Oncol. 2025 Feb 28;30(5):935–43. doi: 10.1007/s10147-025-02726-0 (PMC12014775; doi:10.1007/s10147-025-02726-0)
Supplement: Supplementary file 1 — Supplementary file1 (DOCX 32 KB) [file 10147_2025_2726_MOESM1_ESM.docx]

**Electronic supplemental material**

**Article title:** Pimitespib in patients with advanced gastrointestinal stromal tumors in Japan: an expanded access program

**Journal name:** International Journal of Clinical Oncology

**Author names:** Yoichi Naito, Shiro Iwagami, Toshihiko Doi, Tsuyoshi Takahashi, Yukinori Kurokawa

**Corresponding author:** Yoichi Naito, Affiliation: Department of General Internal Medicine, Medical Oncology, Experimental Therapeutics, National Cancer Center Hospital East, Email: [ynaito@east.ncc.go.jp](mailto:ynaito@east.ncc.go.jp)

## Supplementary Table 1 Baseline patient characteristics in the full analysis set population

|  | **FAS (n=21)** |
| --- | --- |
| Age, years, median (range) | 61.0 (34–77) |
| Sex, n (%) |  |
| Male | 15 (71.4) |
| Female | 6 (28.6) |
| ECOG performance status, n (%) |  |
| 0 | 10 (47.6) |
| 1 | 11 (52.4) |
| Primary tumor, n (%) |  |
| No | 15 (71.4) |
| Yes | 6 (28.6) |
| Primary tumor site, n (%) |  |
| Small intestine | 13 (61.9) |
| Stomach | 7 (33.3) |
| Other | 1 (4.8) |
| Metastases site, n (%) |  |
| Liver | 12 (57.1) |
| Peritoneum | 16 (76.2) |
| Surgery for primary disease, n (%) | 17 (81.0) |
| Therapy for advanced/metastatic disease, n (%) |  |
| Imatinib | 19 (90.5) |
| Sunitinib | 21 (100.0) |
| Regorafenib | 21 (100.0) |
| Other | 4 (19.0) |
| Number of prior systemic anticancer therapies, n (%) |  |
| 3 | 8 (38.1) |
| 4 | 7 (33.3) |
| 5 | 4 (19.0) |
| 6 | 1 (4.8) |
| ≥7 | 1 (4.8) |

ECOG, Eastern Cooperative Oncology Group; FAS, full analysis set

## Supplementary Table 2 Progression-free survival subgroup analysis

|  | **n** | **PFS, median (95% CI)** |
| --- | --- | --- |
| ECOG performance status |  |  |
| 0 | 10 | 3.7 (0.7–NC) |
| 1 | 11 | 4.2 (1.9–NC) |
| Previous lines of anticancer therapy |  |  |
| 3 | 8 | 3.7 (0.7–NC) |
| ≥4 | 13 | 4.2 (1.2–6.2) |
| Age |  |  |
| <65 years | 12 | 4.2 (1.2–NC) |
| ≥65 years | 9 | 4.2 (1.0–NC) |
| Pimitespib dose reduction |  |  |
| Yes | 6 | 4.2 (1.9–NC) |
| No | 15 | 4.2 (1.2–NC) |

CI, confidence interval; ECOG, Eastern Cooperative Oncology Group; NC, not calculated; PFS, progression-free survival.
